# Assessment of peri-neural invasion in oral cancer patients using MRI: A clinical and radiologic correlation study

**DOI:** 10.6026/973206300211118

**Published:** 2025-05-31

**Authors:** Diksha Koushal, Shaivi Sharma, Cheena Singh, Mangala Sajjanar, Prachi Nayak, Swapna Munga, Arpita Saha

**Affiliations:** 1 Department of Oral Medicine and Radiology, Jaipur Dental College, Jaipur, Rajasthan, India; 2 Department of Dentistry, Amaltas Medical College, Dewas, MP, India; 3 Department of Oral Medicine and Radiology, Post Graduate Institute of Dental Sciences, Rohtak, Haryana, India; 4 Department of Oral Pathology, Mallareddy Institute of Dental Sciences, Suraram, Hyderabad, Telangana, India; 5 Department of Oral Pathology, MM College of Dental Sciences and Research, MM (Deemed to be University), Mullana-133207, Haryana, India; 6 Department of Restorative and Prosthetic Dental Sciences, College of Dentistry, King Saud bin Abdulaziz University for Health Sciences, King Abdullah International Medical Research Center, Ministry of National Guard Health Affairs, Riyadh, Kingdom of Saudi Arabia; 7 Intern, Kalinga Institute of Dental Sciences, KIIT Deemed to be University, Patia, Bhubaneswar-751024, Odisha, India

**Keywords:** Perineural invasion, oral cancer, MRI, histopathology, clinical-radiologic correlation

## Abstract

The diagnostic concordance between MRI and histopathology for detecting perineural invasion (PNI) in Oral squamous cell carcinoma (OSCC) patients is of interest. A cross-sectional analytical study was conducted involving 140 patients with histopathologically confirmed OSCC. Demographic, clinical and MRI data were collected, focusing on the presence of PNI, which was compared with histopathological findings for diagnostic accuracy. MRI is a reliable diagnostic tool for detecting perineural invasion in OSCC, with strong agreement to histopathological findings, supporting its clinical utility in treatment planning. Further studies are warranted to validate these findings across larger populations.

## Background:

Oral squamous cell carcinoma (OSCC) remains one of the most prevalent malignancies in the head and neck region, particularly in countries with high tobacco and alcohol consumption [1]. Among the various pathological features that influence prognosis and therapeutic decisions in OSCC, perineural invasion (PNI) has emerged as a critical factor due to its association with increased recurrence, distant metastasis and poor overall survival. PNI is defined as the presence of tumor cells surrounding or invading the nerve sheath and it is considered a marker of aggressive tumor behaviour [2]. The detection of PNI significantly alters treatment planning, often necessitating more aggressive surgical margins and the addition of adjuvant therapies such as radiotherapy or chemoradiation [3]. Traditionally, PNI has been diagnosed through histopathological examination of surgical specimens. However, histology alone may underrepresent the actual extent of perineural involvement due to limitations in sampling [4]. Magnetic Resonance Imaging (MRI) has gained prominence as a valuable non-invasive imaging modality for the detection of soft tissue changes, especially in the perineural space. With superior contrast resolution and multi-planar capabilities, MRI can visualize nerve thickening, obliteration of fat planes and abnormal enhancement patterns, which may suggest PNI even before surgery [5]. This preoperative identification is crucial for risk stratification and for tailoring appropriate oncologic treatment. Despite its potential, the accuracy of MRI in detecting PNI and its correlation with histopathological findings remains a topic of active research [6]. Therefore, it is of interest to evaluate the concordance between MRI-based detection of PNI and histopathological confirmation in oral cancer patients.

## Materials and Methods:

This research was designed as a cross-sectional analytical study involving a total of 140 patients who were histopathologically diagnosed with oral squamous cell carcinoma (OSCC). All patients included in the study had undergone pre-treatment magnetic resonance imaging (MRI) for clinical evaluation. Inclusion criteria mandated that participants must have confirmed OSCC by histopathology and available pre-treatment MRI data. Patients with incomplete clinical or imaging records or those who had received prior oncologic treatment were excluded to maintain data integrity and uniformity. Comprehensive data were collected for each participant. Demographic variables included age, gender and socioeconomic status, classified into low-, middle- and high-income groups. The risk factor profile considered tobacco and alcohol usage, along with a family history of cancer. Tumor-specific characteristics such as the site of the lesion-categorized into buccal mucosa, tongue, alveolus, floor of the mouth and hard palate-were noted. Additionally, each tumor was staged clinically based on the TNM (Tumor-Node-Metastasis) system and graded histopathologically as well, moderately, or poorly differentiated. Details of treatment modalities received by patients were recorded, including surgical intervention, chemoradiation, or a combination of both. The MRI findings specifically assessed the presence of perineural invasion (PNI) and the number of MRI slices analysed. These imaging findings were compared with the histopathological confirmation of PNI to assess diagnostic concordance. For statistical analysis descriptive statistics were used to summarize demographic and clinical variables. Associations between PNI and various factors were tested using the Chi-square test for categorical variables and the t-test for continuous variables. To evaluate the agreement between MRI and histo-pathologic detection of PNI, Cohen's kappa coefficient was calculated. A p-value of <0.05 was considered statistically significant.

## Results:

The Table 1 presents a comprehensive overview of patient demographics and clinical characteristics for a cancer study. The majority of patients were male (68.6%) and aged between 46-60 years (72 patients). Socioeconomic status was skewed toward low (66 patients), and tobacco use was prevalent in 78.6% of patients. Alcohol consumption was reported by 60% of participants. The most common cancer sites were the buccal mucosa (44 patients) and tongue (38 patients), with a significant proportion of cases in advanced stages (Stage III and IV). Histopathologically, most cases were moderately differentiated, with a mix of surgical, chemoradiation, and combined treatment approaches. A substantial number of patients (41.4%) showed positive perineural invasion (PNI) on MRI. Additionally, the average number of MRI slices obtained for the study was 18.4 ± 3.1, providing detailed imaging for evaluation. The Table 2 analyses the association between perineural invasions (PNI) detected through MRI and histopathology (H/P) for various clinical factors. It shows that tobacco use, family history of cancer, advanced cancer stages (III/IV), poor histopathological grade, and cancer site (tongue and floor) are significantly associated with PNI positivity. Specifically, tobacco use and family history of cancer show statistically significant links with PNI on both MRI and H/P (p-value of 0.02 and 0.001, respectively). Additionally, advanced cancer stages (III/IV) and poor histopathological grades strongly correlate with PNI, with p-values of 0.005 and <0.001, respectively. Cancer located in the tongue and floor also exhibited a significant association with PNI positivity, with a p-value of 0.004. These findings suggest that clinical and pathological characteristics, such as tobacco use, family history, cancer stage, and histopathological grade, play an important role in the presence of perineural invasion in patients with head and neck cancers. The Table 3 illustrates the agreement between MRI and histopathological (H/P) findings for perineural invasion (PNI). Out of the 62 histopathology-positive cases, MRI correctly identified 54, yielding a sensitivity of 87.1%. MRI demonstrated a high specificity of 94.9%, correctly identifying 74 out of 78 histopathology-negative cases. The Kappa value of 0.82 indicates a strong agreement between MRI and histopathology for detecting PNI. This suggests that MRI is a reliable imaging modality for identifying perineural invasion, with a high degree of concordance with histopathological results. The Table 4 compares the mean number of MRI slices between PNI-positive and PNI-negative groups. The PNI-positive group had a mean of 20.1 slices (SD = 2.4), while the PNI-negative group had a mean of 17.1 slices (SD = 2.6). The p-value of <0.001 indicates a statistically significant difference between the two groups, suggesting that PNI-positive cases are associated with a higher number of MRI slices. This finding may indicate that more detailed imaging is required to detect perineural invasion, as it appears to be associated with more extensive or complex imaging slices.

## Discussion:

Perineural invasion (PNI) is increasingly recognized as a significant adverse prognostic factor in oral squamous cell carcinoma (OSCC), often correlating with increased locoregional recurrence and reduced survival outcomes. In the present study involving 140 patients with histopathologically confirmed OSCC, PNI was identified in 41.4% of cases on MRI and 44.3% on histopathology, consistent with previously reported incidence rates ranging between 22% and 63% (Chinn & Myers, 2015 [7]; Bakst *et al.* 2019 [8]). The study revealed several critical associations. Tobacco use, a well-known carcinogen, showed a statistically significant correlation with PNI positivity (p = 0.02), suggesting that chronic exposure may enhance neural invasion through tumor-induced neurotrophic factors and inflammatory mediators (Shegaonkar *et al.* 2021) [9]. Similarly, a positive family history of cancer was associated with higher PNI rates (p = 0.001), indicating a possible genetic predisposition or shared environmental exposures that may facilitate neural spread [10]. Notably, patients presenting in advanced stages (III/IV) demonstrated significantly more frequent PNI positivity (p = 0.005). This supports prior findings that PNI often accompanies extensive tumor burden and correlates with aggressive biological behaviour [10, 11]. Moreover, tumors with poor histological differentiation had a markedly higher incidence of PNI (p < 0.001), in line with reports that dedifferentiation is linked to increased invasiveness and nerve infiltration (Manjula *et al.* 2019) [12].

Woolgar *et al.* [13] and Barrett and Speight [6] concluded in their studies that perineural invasion (PNI), whether involving small or large nerves, is associated with increased recurrence and decreased survival rates. Moreover, the prognosis tends to be poorer when PNI affects major nerves, indicating a more aggressive tumor behavior and potentially unfavourable clinical outcomes [14]. Anatomical site also influenced PNI rates. Tumors located in the tongue and floor of mouth showed higher PNI prevalence (MRI PNI: 64.3%, H/P PNI: 67.8%; p = 0.004), potentially due to the rich neural networks in these areas, which may serve as conduits for tumor dissemination [15]. Binmadi and Basile, in their discussion on the significance of perineural invasion (PNI), reported that squamous cell carcinoma (SCC) of the lip exhibited a higher recurrence rate when PNI was present. These cases were notably more challenging to manage and demonstrated a tendency for intracranial spread via regional nerves, leading to subsequent invasion of the central nervous system. This pattern of progression significantly restricted available treatment options and underscored the aggressive nature of such tumors [16, 17]. From a diagnostic perspective, MRI demonstrated high sensitivity (87.1%) and specificity (94.9%) in detecting PNI when compared to histopathology, with a strong kappa agreement of 0.82. This suggests MRI is a reliable non-invasive modality for assessing neural invasion and literature also emphasized the role of high-resolution imaging in OSCC workups [17]. Importantly, the mean number of MRI slices was significantly greater in PNI-positive patients (20.1 ± 2.4 vs. 17.1 ± 2.6; p < 0.001), indicating that meticulous evaluation across more image slices may improve PNI detection accuracy. However, some contrasting studies suggest MRI may still miss microscopic or early PNI, emphasizing the role of histopathology as the gold standard [18]. False negatives can occur due to peritumoral inflammation mimicking or masking neural involvement on imaging. Despite these challenges, this study underlines the complementary value of MRI alongside histopathology, especially in pre-treatment assessment and surgical planning. Alsaffar HA *et al.* (2016) found that both clinical examination and MRI effectively assessed the depth of invasion in oral tongue squamous cell carcinoma when tumors were ≥5 mm deep, but less so for smaller tumors. MRI was more sensitive and specific than clinical assessment. However, clinical evaluation is still valuable in cases where MRI is unavailable or difficult to interpret. Additionally, factors like tumor size, invasion patterns, and perineural or vascular invasion are important for predicting regional metastasis risk [19].

Abdullaeva *et al.* (2024) conducted a meta-analysis on the diagnostic accuracy of MRI in detecting perineural spread (PNS) in head and neck tumors. A total of 11 retrospective studies involving 319 nerve samples from 245 patients were included. The meta-analytic estimates for MRI's performance were as follows: sensitivity 0.85 (95% CI: 0.70-0.95), specificity 0.85 (95% CI: 0.80-0.89), positive predictive value (PPV) 0.86 (95% CI: 0.70-0.94), and negative predictive value (NPV) 0.85 (95% CI: 0.71-0.93). The study revealed significant heterogeneity for sensitivity (I^2^ = 72%, p = 0.003) and PPV (I^2^ = 70%, p = 0.038), but not for specificity or NPV. The most frequent MRI features associated with perineural invasion (PNI) were nerve enlargement and enhancement. Squamous cell carcinoma and adenoid cystic carcinoma were the most common tumor types, with the facial and trigeminal nerves being most commonly affected. Despite some variability in the studies, MRI demonstrated high diagnostic accuracy for detecting perineural invasion in cranial nerves, though evidence was limited and heterogeneous [20]. Valecha, Ojha, Tripathi (2018). MRI plays an essential role in evaluating oral cavity carcinomas, accurately demonstrating key factors such as tumor size, depth of invasion, bone marrow involvement, perineural spread, and lymph node metastasis, all of which are critical for effective treatment planning. This study highlights the significant contribution of MRI in the comprehensive assessment and management of oral cavity cancers [21]. Early identification of PNI can guide more aggressive therapeutic strategies, including wider surgical margins and adjuvant chemoradiation. A key limitation of this study is the potential underdiagnosis of perineural spread (PNS), a macroscopic counterpart of perineural invasion, which may not be fully captured through routine imaging or histopathology, warranting future research focused on advanced molecular and imaging techniques to enhance early detection and improve prognostication.

## Conclusion:

MRI is a reliable modality for detecting perineural invasion in oral cancer, showing strong correlation with histopathologic findings. Its integration in diagnostic protocols can aid in comprehensive treatment planning.

## Figures and Tables

**Table 1:** Dataset summary (n = 140)

**Parameter**	**Category**	**Frequency (%)**
Gender	Male/Female	96 (68.6%)/44 (31.4%)
Age Group	30-45/46-60/>60	28/72/40
Socioeconomic Status	Low/Middle/High	66/56/18
Tobacco Use	Yes/No	110 (78.6%)/30
Alcohol Use	Yes/No	84 (60%)/56
Family History of Cancer	Yes/No	22/118
Cancer Site	Buccal Mucosa/Tongue/Alveolus/Floor/Palate	44/38/26/18/14
Stage (TNM)	I/II/III/IV	14/30/40/56
Histopathological Grade	Well/Moderate/Poor	38/64/38
Treatment Type	Surgery/Chemoradiation/Combined	54/36/50
PNI (MRI)	Positive/Negative	58 (41.4%)/82
PNI (H/P)	Positive/Negative	62/78
MRI Slice Number (Mean±SD)		18.4 ± 3.1 slices

**Table 2:** PNI detection across parameters

**Variable**	**PNI Positive (%) (MRI)**	**PNI Positive (%) (H/P)**	**p-value (Chi-square)**
Gender (Male)	48/96 (50.0%)	50/96 (52.1%)	0.48
Tobacco Use	54/110 (49.1%)	56/110 (50.9%)	0.02*
Alcohol Use	38/84 (45.2%)	40/84 (47.6%)	0.1
Family History	16/22 (72.7%)	18/22 (81.8%)	0.001**
Cancer Stage (III/IV)	48/96 (50.0%)	50/96 (52.1%)	0.005*
Histopath Grade (Poor)	28/38 (73.7%)	30/38 (78.9%)	<0.001***
Site (Tongue & Floor)	36/56 (64.3%)	38/56 (67.8%)	0.004*
Significance: *p<0.05,
*p<0.01, ***p<0.001

**Table 3:** Agreement between MRI and histopathological PNI

**Category**	**H/P PNI Positive**	**H/P PNI Negative**	**Total**
MRI PNI Positive	54	4	58
MRI PNI Negative	8	74	82
Total	62	78	140
Sensitivity (MRI) = 54/62 = 87.1%,
Specificity (MRI) = 74/78 = 94.9%,
Kappa Agreement = 0.82 → Strong Agreement

**Table 4:** Mean MRI Slice Number in PNI

**Group**	**Mean MRI Slices**	**SD**	**p-value (t-test)**
PNI Positive (MRI)	20.1	2.4	<0.001***
PNI Negative	17.1	2.6	

## References

[1] Fatima J (2024). Cureus..

[2] Goswami P.R (2023). Cureus..

[3] Schmitd L.B (2018). J Dent Res..

[4] Bahmad H.F (2023). Curr Oncol..

[5] Börnert P (2020). Br J Radiol..

[6] Barrett A.W, Speight P (2011). Curr Cancer Ther Rev..

[7] Chinn S.B, Myers J.N (2015). J Clin Oncol..

[8] Bakst R.L (2019). Int J Radiat Oncol Biol Phys..

[9] Shegaonkar A (2021). J Evol Med Dent Sci..

[10] Dhawan A (2018). J Maxillofac Oral Surg..

[11] Fagan J.J (1998). Arch Otolaryngol Head Neck Surg..

[12] Manjula M (2019). J Oral Maxillofac Pathol..

[13] Woolgar J.A (2006). Oral Oncol..

[14] Barrett A.W, Speight P.M (2009). Oral Oncol..

[15] Deepthi G (2020). J Oral Maxillofac Pathol..

[16] Binmadi N.O, Basile J.R (2011). Oral Oncol..

[17] Medvedev O (2022). Bosn J Basic Med Sci..

[18] Sharma P (2023). J Neurol Surg B Skull Base..

[19] Alsaffar H.A (2016). J Otolaryngol Head Neck Surg..

[20] Abdullaeva U (2024). Diagnostics (Basel)..

[21] Valecha J (2018). Int J Med Res Rev..

